# Cation-cation clusters in ionic liquids: Cooperative hydrogen bonding overcomes like-charge repulsion

**DOI:** 10.1038/srep17505

**Published:** 2015-12-02

**Authors:** Anne Knorr, Ralf Ludwig

**Affiliations:** 1 Universität Rostock, Institut für Chemie, Abteilung für Physikalische Chemie, Dr.-Lorenz-Weg 1, 18059, Rostock (Germany); 2 Leibniz-Institut für Katalyse an der Universität Rostock e.V., Albert-Einstein-Str. 29a, 18059 Rostock (Germany)

## Abstract

Direct spectroscopic evidence for H-bonding between like-charged ions is reported for the ionic liquid, 1-(2-hydroxyethyl)-3-methylimidazolium tetrafluoroborate. New infrared bands in the OH frequency range appear at low temperatures indicating the formation of H-bonded cation-cation clusters similar to those known for water and alcohols. Supported by DFT calculations, these vibrational bands can be assigned to attractive interaction between the hydroxyl groups of the cations. The repulsive Coulomb interaction is overcome by cooperative hydrogen bonding between ions of like charge. The transition energy from purely cation-anion interacting configurations to those including cation-cation H-bonds is determined to be 3–4 kJmol^−1^. The experimental findings and DFT calculations strongly support the concept of anti-electrostatic hydrogen bonds (AEHBs) as recently suggested by Weinhold and Klein. The like-charge configurations are kinetically stabilized with decreasing temperatures.

The association of ions plays an important role for reactions in solution, macromolecular catalysis, biochemical hydrolysis and protein stability^1^,^2^,^3^. Whereas the concept of pairing between opposite-charge ions is well accepted, the association of like-charged ions seems to be a counter-intuitive phenomenon. Only little experimental evidence is reported for this elusive concept. Like-charge attraction has been observed only for aqueous salt solutions K/CsBr^4^, for guanidinium ions in water^5^, for the micellation of tetraalkylammonium surfactants^6^, and for metastable colloidal crystallites^7^. In all cases the attractive interaction between like-charged ions is mediated by the solvent molecules, mostly water. Like-charge attraction is also reported for biomolecular systems including oligopeptides and DNA^8^,^9^. For ionic liquids (ILs), consisting purely of cations and anions, Mele *et al.* reported NOE contacts between protons of imidazolium cations^10^. However, Weingärtner and Steinhauser *et al.* challenged these results. They argue that Mele’s analysis was only taken in the spirit of the intramolecular NOE and thus ignores the intermolecular part which is strongly influenced by the dynamics of the spin-pairs^11^,^12^. Most recently, Gamrad *et al.* could report self-association of simple organic cations based on hydrogen bonding. Cation-cation pairing in crystal structures could be detected by means of X-ray diffraction^13^. This type of “anti-electrostatic” hydrogen bonds (AEHBs) was recently suggested by Weinhold and Klein based on ab initio and hybrid density functional calculations^14^. They characterized a surprising new class of H-bonded complexes comprised by ions of like charge. It was claimed that these species exhibit appreciable ‘kinetic stability’ and typical structural and spectroscopic signatures of hydrogen bonding, despite strong repulsive electrostatic forces. However, the calculated effects were small and most of the systems are not well suited for experiment. Moreover, this concept was strongly opposed by Frenking and Caramori^17^,^18^. To the best of our knowledge, proper spectroscopic evidence and analysis for attractive interactions between ions of like charge in ionic liquids could not be reported so far.

In principle, cation-cation attraction in ILs should be possible by substantial charge delocalisation in the cation and the use of weakly coordinating anions^19^,^20^,^21^. Thus, for studying and characterizing anti-electrostatic hydrogen bonds we have chosen the ionic liquid 1-(2-hydroxyethyl)-3-methylimidazolium tetrafluoroborate [HEMIm][BF_4_] for two reasons. Firstly, the interacting hydroxyl group of the imidazolium cation is away from the center of positive charge and secondly, the tetrafluoroborate is a weakly interacting anion. Both features promise possible directional, cooperative H-bonds between the cations overcoming the repulsive electrostatic forces. In this work, we report spectroscopic evidence for AEHBs in pure ionic liquids. The experimental findings are fully supported by DFT calculations on differently sized ion-pair clusters including opposite-charge and like-charge attraction only. Although slightly higher in energy, the configurations including H-bonded cations are kinetically stable. The attractive interaction between charge-like ions is expected to have strong influence on the structure and dynamics of ionic liquids.

The infrared spectra for the O-H stretches of the ionic liquid [HEMIm][BF_4_] are shown in Fig.1a as a function of temperature between 233 K and 413 K. All IR spectra could be properly decomposed into four vibrational bands (see SI). At the highest temperature, a main OH vibrational band is observed at 3561 cm^−1^, which can be clearly assigned to the expected OH^…^F hydrogen bond between cation and anion. The high wavenumber indicates that the cation-anion hydrogen bond is relatively weak. It can be related to the interaction strength of water and methanol in similar ionic liquids including BF_4_^−^ anions^22^,^23^. However, the vibrational band is not symmetric and exhibits additional intensity at the low frequency site. With decreasing temperature, this intensity initially results in a broad shoulder and finally in a distinct vibrational band at about 3402 cm^−1^ at 233 K. At the same time the main vibrational band at 3561 cm^−1^ is slightly redshifted by about 14 cm^−1^. With decreasing temperature, usually H-bonds become stronger, resulting in further redshift but also in increasing intensity. However, below 303 K the intensity of the main band at 3561 cm^−1^ decreases for the benefit of a new band at 3402 cm^−1^, indicating a shift of intensity from one species to the other. The latter vibrational band is redshifted by about 145 cm^−1^ relative to the dominant OH band at 3547 cm^−1^ at the lowest temperature. Such a strong redshift can be only assigned to different classes of bound OH groups. Overtone or combination bands can be excluded in this frequency range. Fig. 1b shows that the lowest temperature spectrum (233 K) can be decomposed into four contributions at 3547 cm^−1^, 3496 cm^−1^, 3402 cm^−1^ and 3286 cm^−1^, respectively (also see SI).

For the assignment of the deconvoluted bands of the measured spectra we performed DFT calculations at the B3LYP/6-31+G* level of theory including D3 dispersion correction on different sized clusters up to four ion-pairs (n = 1–4) for this IL^24^,^25^,^26^. Two general classes of H-bonding configurations were considered. In structures **ca** with n = 1–4, only hydrogen bonding of the hydroxyl group of the cation with the anion (OH^…^F) is present resulting in frequencies assigned to the main vibrational band in the measured IR spectra at 3561 cm^−1^ (413 K) and at 3547 cm^−1^ (233 K). In structures **cc** with n = 2–4 additional hydrogen bonding between the hydroxyl groups of the cations (OH^…^OH, like-charge interaction) is present. For n = 4 two **cc** configurations were considered: in linear **cc** the cations form linear clusters with the end-standing OH interacting with the anion, in **cc-cyc** they form cyclic clusters without any H-bond to the anions. Both species, the linear as well as the cyclic tetramers are also stable as fourfold charged cations. As an example for the different classes of H-bonded clusters, all geometry optimized IL tetramers are shown in Fig. 2 (see SI).

Only real frequencies are found indicating true minimum structures on the potential energy surface. The energetic features of these clusters will be discussed later. The calculated OH vibrational frequencies for all species **ca** and **cc** are shown in the Supporting Information. The frequencies of the **ca**-species n = 1–4 span a spectral range of maximal 45 cm^−1^. Such a narrow frequency distribution indicates moderately different H-bonds between cation and anions (OH^…^F). Part of this frequency range is covered by the measured temperature shift of the main vibrational band of about 14 cm^−1^. The calculated OH stretch frequencies of the **cc**-species are redshifted throughout compared to the frequencies for the **ca**-species. The calculated redshifts of the average OH vibrational frequencies for each cluster between the **ca** and **cc** species are shown in Fig. 3.

Overall, three main results can be reported: Firstly, the vibrational bands of the end-standing OH interacting with the BF_4_^−^ anion in the linear cc-species, is only slightly redshifted due to cooperative effects (~20 cm^−1^). This small shift falls into the range covered by the differently bound **ca** species in n = 1–4. Secondly, the smallest **cc**-species (n = 2) show only weak redshifts compared to the **ca** bands (Δν = 41 cm^−1^). These species can account for the asymmetry of the main band but not for the distinct measured contribution at 3402 cm^−1^. The calculated OH frequencies of **cc**-species for n = 3,4 are significantly stronger shifted to lower wavenumbers. As well known for water and alcohol clusters, H-bonds are enhanced due to cooperative effects resulting in weakened OH bonds and redshifted OH frequencies^27^,^28^,^29^,^30^,^31^. No further redshift can be observed from the **cc** trimer to the **cc** tetramer indicating that cooperativity is saturated for the linear trimer species already. The calculated redshifts for **cc** trimer and the **cc** tetramer are found to be 165 cm^−1^ and 145 cm^−1^, respectively. Such a small difference in frequency cannot be properly resolved in our measured spectra, but both frequencies are close to that of the deconvoluted band at 3402 cm^−1^ (Δν = 145 cm^−1^). Thirdly, the cyclic cationic cluster with a calculated redshift of about 260 cm^−1^ is also present in the liquid. The deconvoluted band at 3286 cm^−1^ leads to a redshift of about 259 cm^−1^, in perfect agreement with the predicted shift from the DFT-D3 calculated cyclic tetramer. Overall, it is shown in Fig. 3 that all the redshifts of the deconvoluted vibrational bands from the measured spectra (grey bars) reasonably agree with those of the linear dimers, trimers and tetramers (open circles) as well as the cyclic tetramer (open square). We also added the redshifts (dotted brownish line) obtained from the calculated derivatives of the measured spectra supporting all the deconvoluted bands except the dimer (see SI). Overall, it can be claimed that the redshifted vibrational bands stem from OH^…^OH interaction between ions of like charge. For the first time we can report spectroscopic evidence for directional cation-cation interaction via hydrogen bonding in ILs. Moreover, the vibrational bands can be assigned to linear and cyclic species of different size as known for water and alcohols.

As shown in Fig. 1b, the measured spectra could be deconvoluted into one vibrational band assigned to the OH stretch of the **ca**-species and three redhifted bands all related to the **cc**-species, dimers, trimers and tetramers (see SI). If we assume that the cation-anion (I_ca_) and the cation-cation (I_cc_) hydrogen bond intensities arise from cations in two general classes of H-bonding configurations (**ca**: OH bound to the anion and **cc**: OH bound the cation), for which relative populations are a function of absolute temperature only, a plot of ln(I_cc_/I_ca_) versus 1/T will yield a straight line with a slope that is proportional to the average difference in energy between the two classes (ΔE). A linear fit of the data yields a correlation coefficient of 0.98 and a slope ΔE/R, where R is the universal gas constant, that gives the transfer energy between the two classes of H-bonding distribution as 2.9 ± 0.02 kJmol^−1^ (Fig. 4). However, the absorption coefficients for the vibrational bands of the **ca** and **cc** species may be different. If we weight the measured absorption by the DFT-D3 calculated intensities for each species, we obtain a little higher transfer energy of about 3.4 ± 0.02 kJmol^−1^. In a third procedure, we calculated I_cc_ and I_ca_ from the integral intensities left and right of the frequency position, where the deconvoluted vibrational bands for the **ca** and the first **cc** species (dimer) cross (see SI). Here, we calculated ΔE to be 3.9 ± 0.02 kJmol^−1^. Whatever procedure we use the transfer energies range between 2.9 and 3.9 kJmol^−1^. We would like to emphasize that the second approach seems to be the most reliable (ΔE = 3.4 ± 0.02 kJmol^−1^). Obviously, both configurations only differ slightly in energy. In the **cc**-species, the repulsive Coulomb interaction between the cations can be overcome by attractive and cooperatively enhanced H-bond interaction.

Transition energies can be also estimated from the differences of the DFT-D3 (B3LYP/6-31+G*) calculated energies and free energies of the **ca**- and **cc**-clusters (see SI). For example, the tetramers **ca** are slightly favoured in energy over the **cc**- and **cc-cyc**-structures by about 5.83 kJmol^−1^ and 1.0 kJmol^−1^ in energy per ion pair. For the free energies these ratios become even more favourable for the **cc**-species (ΔG  =  2.96 kJmol^−1^ and 0.2 kJmol^−1^). Although the choice of **ca**- and **cc**-clusters is somewhat arbitrary, the calculated differences in energy and free energy are in the right magnitude and support the interpretation of the experimental data. Overall, the calculated energies suggest that the **cc** configurations are kinetically stabilized at low temperatures and present in equilibrium. Here, we would like to point out that the cation-cation clusters are not only stable due to the presence of the BF_4_^−^ anions. In fact, the DFT-D3 calculations show that H-bonding overcomes the repulsive Coulomb forces already for the purely cationic clusters (see SI).

What is the cause of the attractive forces between the like-charged ions? The B3LYP-6-31+G*-D3 calculations suggest that the cation-cation OH^…^OH interaction is possible due to cooperative effects^30^,^31^. Charge from the anion fluorine is donated into the OH anti-bond of the first cation. The larger negative charge at the OH oxygen can now be transferred into the OH anti-bond of the second cation further enhancing hydrogen bonding. This process leads to even stronger cooperativity in the trimers and tetramers. This way the short-range donor-acceptor covalency forces can overcome the strong long-range electrostatic repulsive forces as expected for ions of like charge. These features can be rationalized in the framework of the natural bond orbital (NBO) analysis^15^,^16^,^17^,^32^. The repulsive electrostatic forces are overcome by directional, cooperative H-bonds indicated by characteristic redshift in the OH stretch region. NBO analysis shows typical strong n_o_ → σ*_OH_ donor-acceptor interaction, corresponding to second order stabilization energies Δ*E*(2)_n→σ*_ and estimated total charge transfers of *q*_TC_ for the F^…^OH as well as for the enhanced OH^…^OH hydrogen bonds, respectively. For both properties, we plotted the differences between the **cc**- and **ca**-clusters versus the calculated redshifts. Both, the differences in stabilization energies and in charge transfer reflect the order of the IR redshifts as shown in Fig. 5 (see also SI). That there is no further redshift from the linear trimer to the linear tetramer is confirmed by saturation or even decreasing of Δ(Δ*E*(2)_n→σ*_) and Δ(*q*_CT_). However, the strongest intermolecular stabilization energy is found for the cyclic tetramer and is well correlated with the cooperative binding energies and frequencies. The ‘closed-CT’ networks have highest stability resulting in strongest interaction and largest vibrational red shifts. Here, we find the same behaviour for H-bonded cations as reported for H-bonded molecules such as water or alcohols^33^,^34^.

For the first time we could provide evidence for hydrogen bonding between ions of like charge in ionic liquids. In principle the hydroxyl groups of the cations can form cooperative H-bonds OH^…^OH similar to those in water or alcohol clusters. The like-charge attraction is governed by a delicate balance of Coulomb repulsion and cooperative hydrogen bonding well beyond the simple pairwise additive interaction. Thus, enhanced H-bonding can overcome the repulsive Coulomb forces representing AEHBs as suggested recently by Weinhold and Klein. We show that the like-charge clusters are slightly disfavoured in energy but kinetically stabilized at low temperatures. In future studies we would like to show how like-charge attraction depends on the type of cation and on the interaction potential of the chosen anions. That should allow the control of important IL properties such as viscosities and conductivities. These kinds of studies are currently going on in our laboratories.

## Additional Information

**How to cite this article**: Knorr, A. and Ludwig, R. Cation-cation clusters in ionic liquids: Cooperative hydrogen bonding overcomes like-charge repulsion. *Sci. Rep.* **5**, 17505; doi: 10.1038/srep17505 (2015).

## Supplementary Material

Supplementary Information

## Figures and Tables

**Figure 1:**
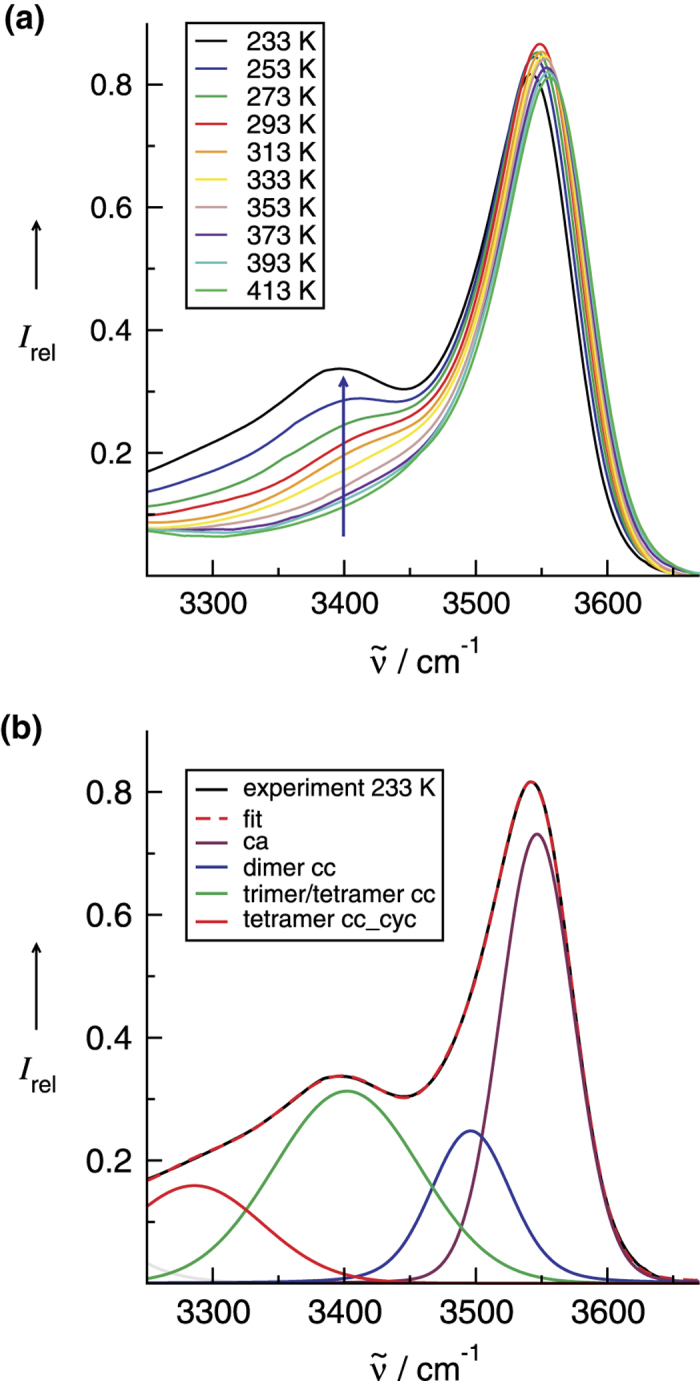
(**a**) Infrared spectra of the O-H stretching vibrations of the ionic liquid 1-(2-hydroxyethyl)-3-methylimidazolium tetrafluoroborate as a function of temperature between 233 K and 413 K. The arrow indicates the increasing intensitiy of the appearing vibrational band at 3402 cm^−1^. (**b**) The IR spectrum at the lowest temperature (233K) can be properly decomposed into four contributions at 3547 cm^−1^, 3496 cm^−1^, 3402 cm^−1^ and 3286 cm^−1^, respectively.

**Figure 2:**
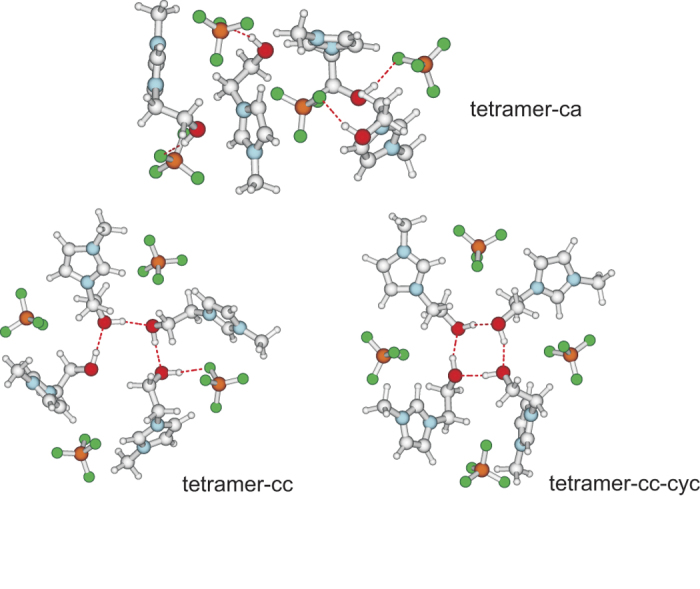
B3LYP-6-31G*-D3 calculated structures of possible tetrameric clusters including four ion-pairs: tetramer ca, including solely cation-anion pairs characterized by OH^…^F hydrogen bonds; tetramer cc, with additional cation-cation interaction resulting in cooperative hydrogen bonding OH^…^OH^…^OH^…^OH^…^F; and tetramer **cc-cyc**, showing a strongly cooperative H-bonded cyclic tetramer of cations via (OH^…^O)_4_ bonds.

**Figure 3:**
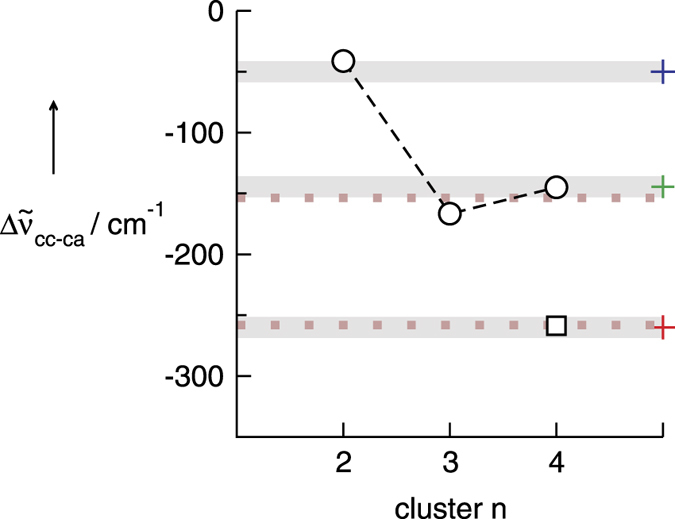
A plot of the calculated O-H vibrational redshifts for all cc-clusters: the linear dimer, trimer and tetramer (open circles) and the cyclic tetramer (open square). The shifts are determined relative to the average of the O-H stretches of the corresponding **ca**-clusters n = 2–4. For comparison, the redhifts of the deconvoluted bands of the measured spectra are shown as grey bars (the colors of the crosses indicate the deconvoluted bands in Fig. 1b). The calculated redshifts strongly suggest that all species are present in the liquid. The dotted lines indicate the frequency shifts obtained from calculating the derivatives of the measured spectra (see SI).

**Figure 4:**
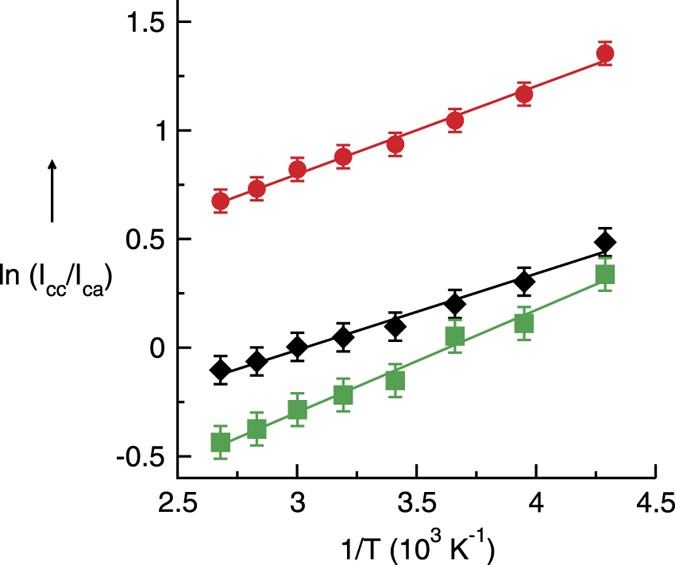
Plots of the natural logarithm of the cc to ca vibrational band intensity ratios versus inverse temperature, taken from the measured spectra in Fig. 1 between 233 K and 373 K. Three procedures were applied: (**a**) I_cc_ and I_ca_ of the deconvoluted bands (diamonds), (**b**) I_cc_ and I_ca_ of the deconvoluted bands each weighted by the calculated intensities of the species (circles) and (**c**) I_cc_ and I_ca_ obtained from the integral intensities left and right of the frequency position, where the deconvoluted vibrational bands for the **ca** and the first **cc** species cross (squares). The solid lines represent linear fits (R^2^ ≥ 0.98) with a slope of ΔE/R. ΔE, the difference in energy between the two different H-bonding configurations, is determined to be (a) 2.9 ± 0.02 kJmol^−1^, (**b**) 3.4 ± 0.02 kJmol^−1^ and (**c**) 3.9 ± 0.02 kJmol^−1^, respectively.

**Figure 5:**
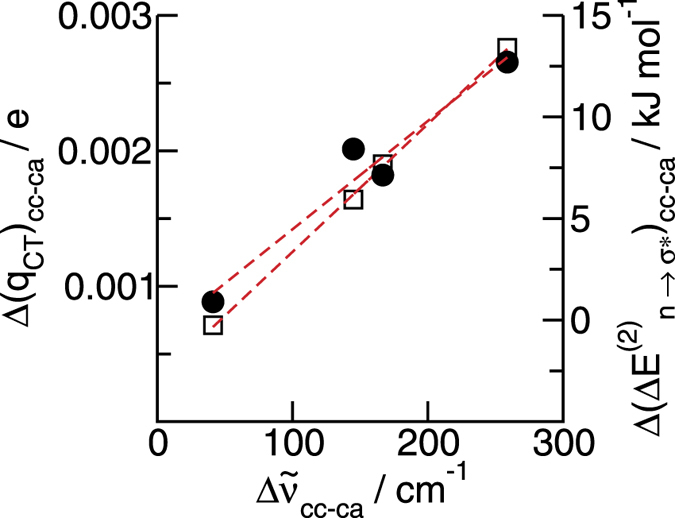
Differences of NBO calculated stabilization energies ΔE(2)_n→σ*_ (circles) and estimated total charge transfers q_TC_ (squares) between the clusters n = 2–4 for the two classes of H-bond configurations **cc** and **ca**, plotted versus calculated average OH redshifts. The linear dependence indicates the strong relation between NBO stabilization energies and charge transfers with spectroscopic properties such as IR frequencies.
