# Supplementary material for: Cation-cation clusters in ionic liquids: Cooperative hydrogen bonding overcomes like-charge repulsion
Source: Sci Rep. 2015 Dec 2;5:17505. doi: 10.1038/srep17505 (PMC4667241; doi:10.1038/srep17505)
Supplement: Supplementary Information [file srep17505-s1.pdf]

# Cation-cation clusters in ionic liquids: Cooperative hydrogen bonding overcomes like-charge repulsion*

Anne Knorr and Ralf Ludwig[a,b]

[a]Dipl.-Chem. Anne Knorr, Prof. Dr. R. Ludwig
Universität Rostock, Institut für Chemie, Abteilung für Physikalische Chemie
Dr.-Lorenz-Weg 1, 18059, Rostock (Germany)
Tel: (+49) 381-498-6517
E-mail: ralf.ludwig@uni-rostock.de

[b]Prof. Dr. R. Ludwig
Leibniz-Institut für Katalyse
an der Universität Rostock e.V.
Albert-Einstein-Str. 29a, 18059 Rostock (Germany)

## Supplementary Information

**Contents**

**SI0** **Sample preparation**

**SI1** **Experimental**

**SI2** **Deconvoluted mid infrared (MIR) spectra of [HEMim][$BF_4$] between 233 K and 413 K**

**SI3** **Intensities of the deconvoluted vibrational bands of the MIR spectra of [HEMim][$BF_4$] between 233 K and 413 K**

**SI4** **DFT-D3 optimized geometries of [HEMim][$BF_4$] clusters ca and cc**

**SI5** **DFT-D3 calculated frequencies of [HEMim][$BF_4$] clusters ca and cc**

**SI6** **DFT-D3 calculated energies, free energies and NBO analysis of [HEMim][$BF_4$] clusters ca and cc**

## SI0 Sample preparation

The sample of [HEMim][$BF_4$] was purchased from Iolitec (>99% mass fraction). The ionic liquids were dried under vacuum for around 48 hours in order to reduce the water content under 100 ppm and remove other volatile impurities as well. The water content was checked using a Karl Fischer titrator (Titroline KF Trace, Schott Instruments GmbH) resulting in 64 ppm.

## SI1 Experimental

Mid infrared (MIR) measurements were performed with a Bruker Vector 22 FTIR spectrometer. An L.O.T.-Oriel variable-temperature cell equipped with $CaF_2$ windows having a path length of 12 μm was used for the variable-temperature experiments between 233 and 413 K. Cooling of the cell is achieved by means of a cooling dewar with liquid ethanol/nitrogen mixture. For each spectrum 128 scans were recorded at a spectral resolution of 1 $cm^{-1}$.

The spectra were deconvoluted simultaneously as well as separately into a number of Voigt-profiles (convolution of Lorentzian and Gaussian functions) following the Levenberg-Marquardt procedure. The Voigt-profile has four parameters: the intensity, the frequency, the half-width of the Lorentzian, and the half-width of the Gaussian.

## SI2a Deconvoluted mid infrared (MIR) spectra of [HEMim][$BF_4$] between 233 K and 413 K

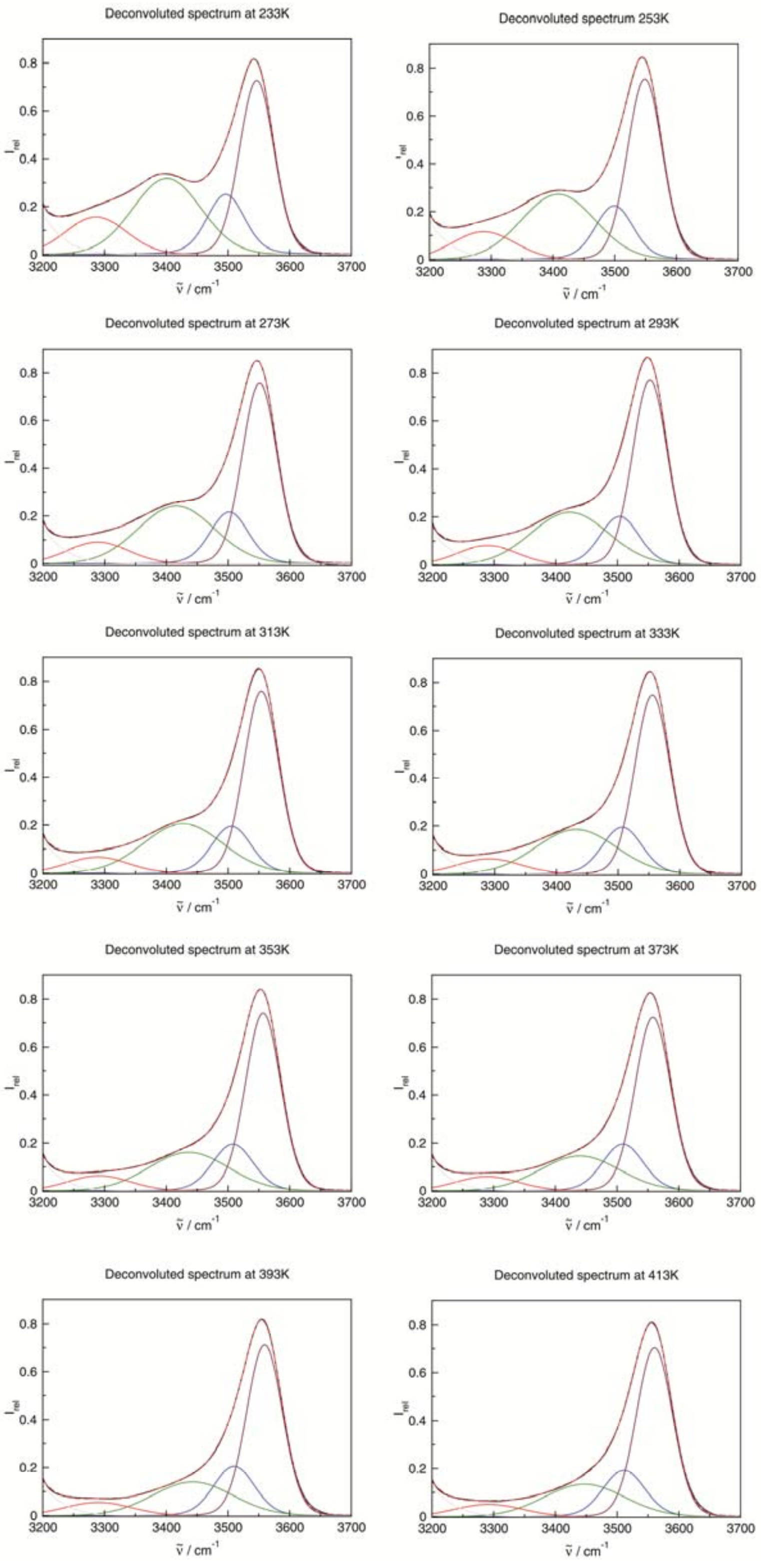


**SI-FIG1a** Deconvoluted MIR spectra of [HEMim][$BFf_4$] as a function of temperature. The spectra are shown for the frequency range between 3200 and 3700 $cm^{-1}$.

**SI2a Derivatives of the mid infrared (MIR) spectrum of [HEMim][$BF_4$] at 233 K**

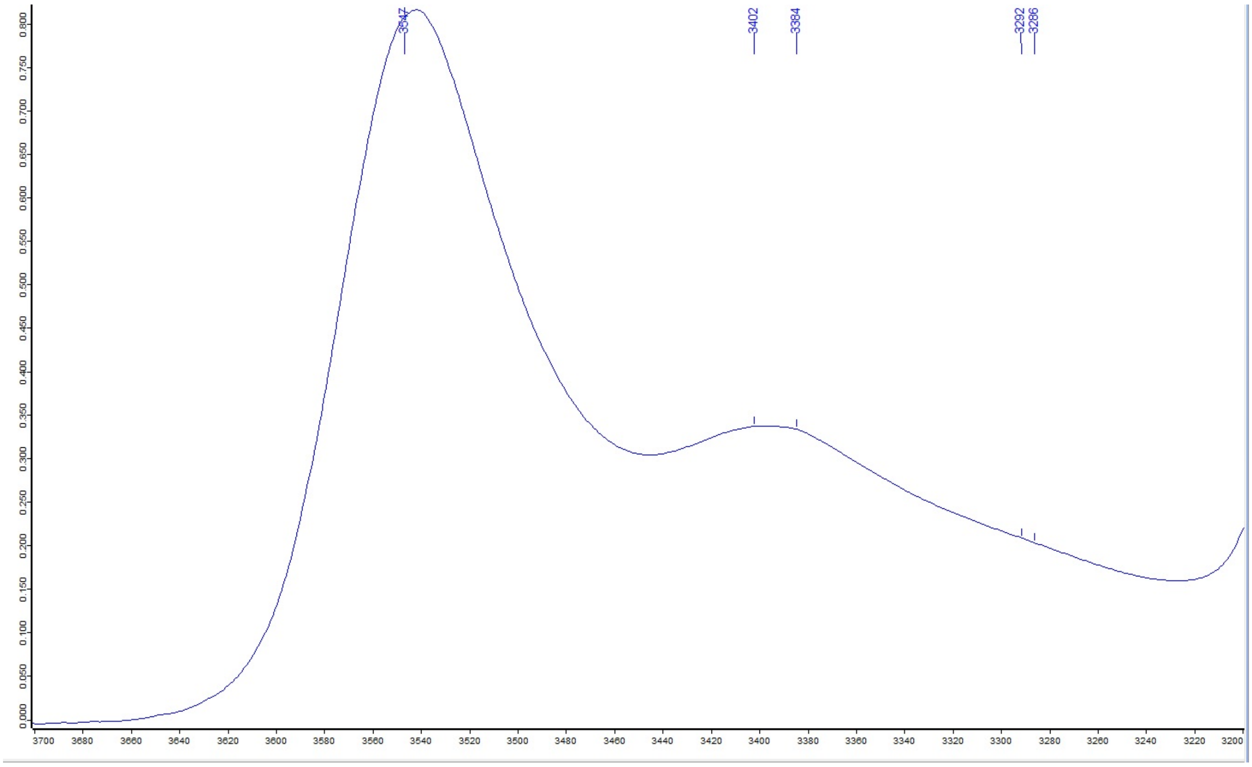


**SI-FIG1b** Derivatives of the MIR spectrum of [HEMim][$BFf_4$] at 233 K. The derived frequencies are located at the positions where we find the **ca** structure and the **cc** linear trimers, tetramers and cyclic tetramer.

## SI3 Intensities of the vibrational bands deconvoluted from the mid infrared (MIR) spectra of [HEMim][$BF_4$] between 233 K and 413 K

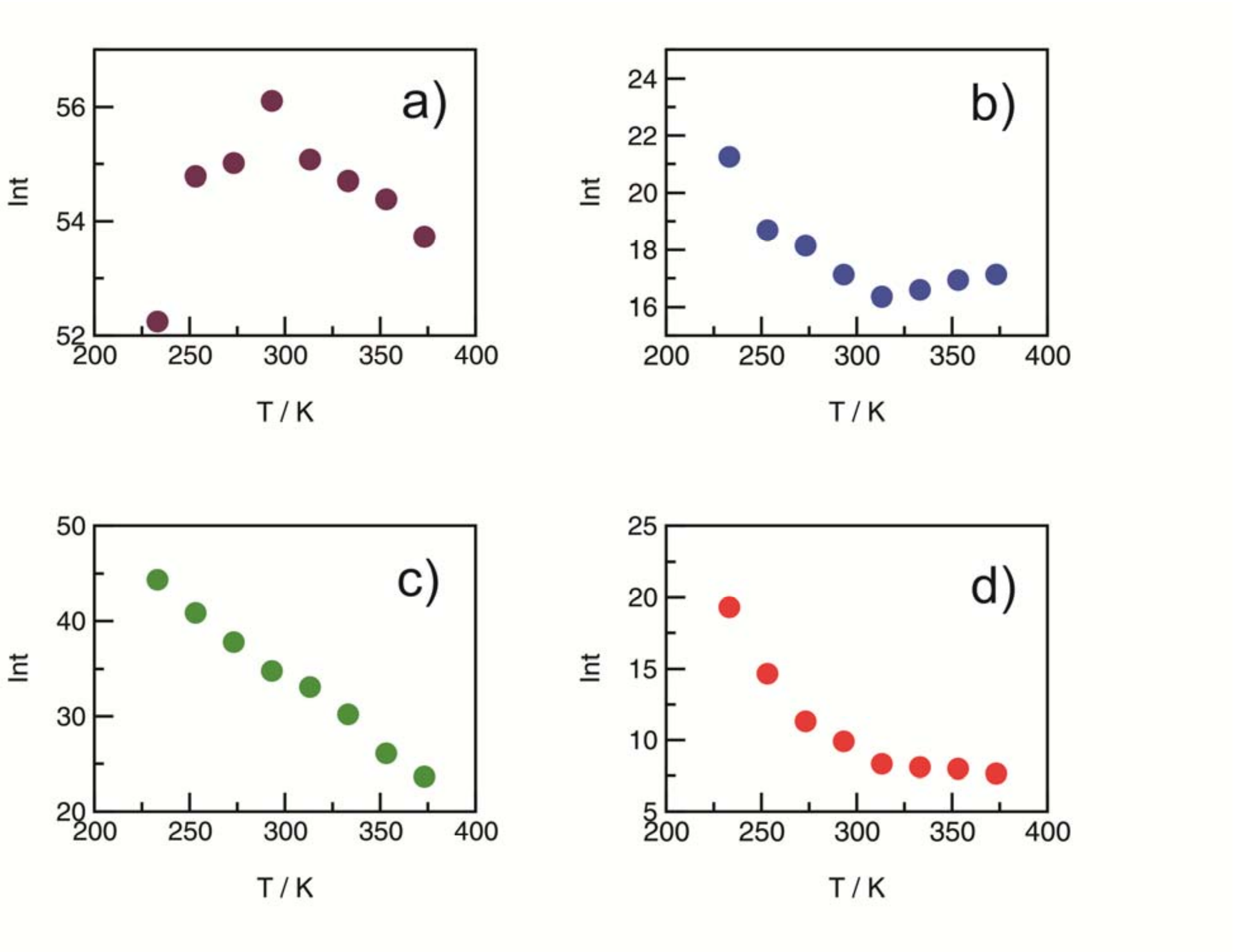


**SI-FIG2** Intensities of the vibrational bands deconvoluted from the MIR spectra of [HEMim][$BF_4$] as a function of temperature.
a) ca, b) dimer cc, c) trimer, tetramer cc, and d) tetramer cyc.

All spectra were deconvoluted separately into a number of Voigt profiles (convolution of Lorentzian and Gassian functions) following the Levenberg-Marquardt procedure. The Voigt profile has four parameters: the intensity, the frequency, the half-width of the Lorentzian, and the half-width of the Gaussian. The frequency and the half-widths of each Voigt function for all vibrational modes were kept fixed in the simultaneous fitting procedure.

## SI4 DFT-D3 optimized geometries of [HEMim][$BF_4$] clusters ca and cc

The IL clusters have been calculated at the DFT level B3LYP, using the internal stored 6-31+G* basis set of the Gaussian 09 program.[24] Grimme's DFT-D3 method was applied for calculating dispersion forces.[25,26]

## Literature


[24] Gaussian 03, Revision C.02, M. J. Frisch, G. W. Trucks, H. B. Schlegel, G. E. Scuseria, M. A. Robb, J. R. Cheeseman, J. A. Montgomery, Jr., T. Vreven, K. N. Kudin, J. C. Burant, J. M. Millam, S. S. Iyengar, J. Tomasi, V. Barone, B. Mennucci, M. Cossi, G. Scalmani, N. Rega, G. A. Petersson, H. Nakatsuji, M. Hada, M. Ehara, K. Toyota, R. Fukuda, J. Hasegawa, M. Ishida, T. Nakajima, Y. Honda, O. Kitao, H. Nakai, M. Klene, X. Li, J. E. Knox, H. P. Hratchian, J. B. Cross, V. Bakken, C. Adamo, J. Jaramillo, R. Gomperts, R. E. Stratmann, O. Yazyev, A. J. Austin, R. Cammi, C. Pomelli, J. W. Ochterski, P. Y. Ayala, K. Morokuma, G. A. Voth, P. Salvador, J. J. Dannenberg, V. G. Zakrzewski, S. Dapprich, A. D. Daniels, M. C. Strain, O. Farkas, D. K. Malick, A. D. Rabuck, K. Raghavachari, J. B. Foresman, J. V. Ortiz, Q. Cui, A. G. Baboul, S. Clifford, J. Cioslowski, B. B. Stefanov, G. Liu, A. Liashenko, P. Piskorz, I. Komaromi, R. L. Martin, D. J. Fox, T. Keith, M. A. Al-Laham, C. Y. Peng, A. Nanayakkara, M. Challacombe, P. M. W. Gill, B. Johnson, W. Chen, M. W. Wong, C. Gonzalez, and J. A. Pople, Gaussian, Inc., Wallingford CT, 2004.

[25] S. Grimme, J. Antony, S. Ehrlich, H. Krieg, *J. Chem. Phys.* **2010**, 132, 154104.

[26] S. Ehrlich, J. Moellmann, W. Reckien, T. Bredow, S. Grimme**,** ChemPhysChem. **2011**, 12, 3414-3420.

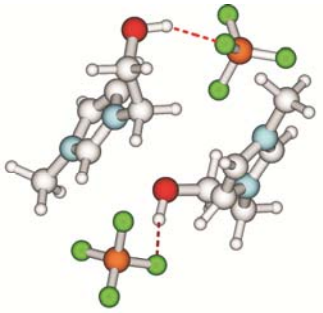

dimer-ca

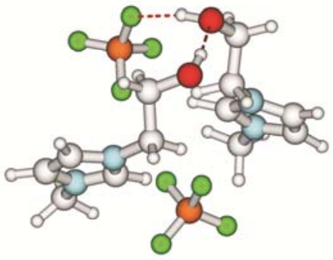

dimer-cc

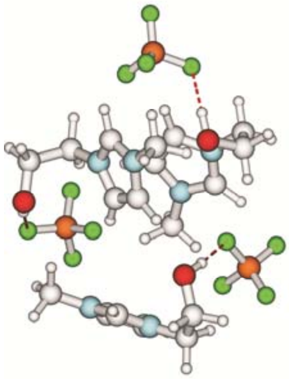

trimer-ca

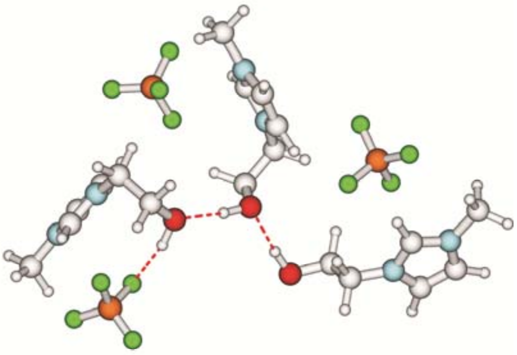

trimer-cc

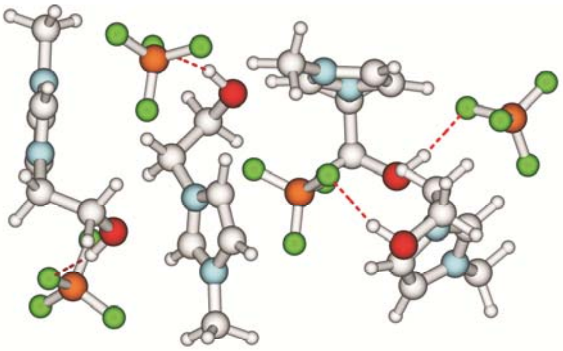

tetramer-ca

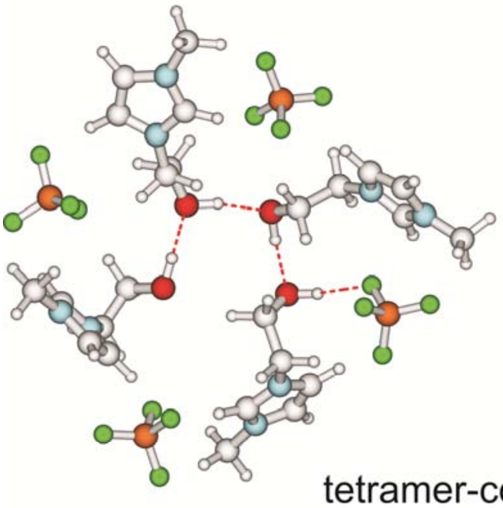

tetramer-cc

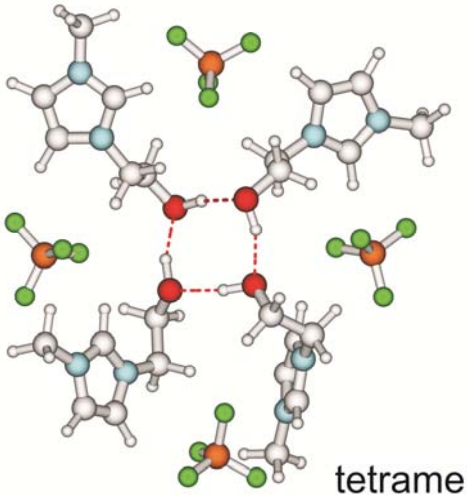

tetramer-cc-cyc

**dimer ca**

*** imOH_BF4_b3lyp_6-31+Gp_dimer_D3.g09, -1689.04027659

| | | | | |
|---|---|---|---|---|
| 7 | 0 | -0.537230 | 2.319948 | -0.849060 |
| 6 | 0 | 0.079166 | 2.495557 | 0.378231 |
| 6 | 0 | -0.912544 | 2.535375 | 1.313038 |
| 7 | 0 | -2.116215 | 2.375168 | 0.644149 |
| 6 | 0 | -1.861669 | 2.239428 | -0.660930 |
| 6 | 0 | -3.445624 | 2.364704 | 1.264570 |
| 6 | 0 | 0.153948 | 2.190193 | -2.143103 |
| 6 | 0 | 0.471964 | 0.736349 | -2.516030 |
| 9 | 0 | -3.396662 | -0.575774 | 0.785971 |
| 5 | 0 | -3.434495 | -0.673529 | -0.651724 |
| 9 | 0 | -4.179467 | 0.420674 | -1.145587 |
| 9 | 0 | -2.091476 | -0.547555 | -1.110951 |
| 9 | 0 | -3.962621 | -1.893346 | -1.025618 |
| 9 | 0 | 3.063705 | 1.887146 | -0.557436 |
| 5 | 0 | 3.664061 | 1.276807 | 0.610635 |
| 9 | 0 | 4.605071 | 2.117429 | 1.155981 |
| 9 | 0 | 4.213600 | 0.036264 | 0.207973 |
| 9 | 0 | 2.601133 | 1.015687 | 1.527377 |
| 6 | 0 | 1.839458 | -1.829425 | 0.462156 |
| 7 | 0 | 0.510647 | -1.835911 | 0.598695 |
| 6 | 0 | -0.031927 | -2.712530 | -0.323312 |
| 6 | 0 | 1.013750 | -3.251248 | -1.013232 |
| 7 | 0 | 2.173859 | -2.687739 | -0.505817 |
| 6 | 0 | -0.233465 | -0.942558 | 1.494133 |
| 6 | 0 | -0.991614 | -1.707895 | 2.581816 |
| 6 | 0 | 3.531223 | -2.903695 | -1.006543 |
| 1 | 0 | -0.481997 | 2.643849 | -2.909662 |
| 1 | 0 | 1.072707 | 2.777098 | -2.063693 |
| 1 | 0 | -3.462103 | 1.608232 | 2.049623 |
| 1 | 0 | -3.658887 | 3.356107 | 1.672803 |
| 1 | 0 | -4.180338 | 2.085988 | 0.510694 |
| 1 | 0 | -2.604360 | 2.033660 | -1.415881 |
| 1 | 0 | -0.867805 | 2.654890 | 2.384243 |
| 1 | 0 | 1.152987 | 2.533363 | 0.473610 |
| 1 | 0 | 3.855583 | -3.922107 | -0.775778 |
| 1 | 0 | 3.538433 | -2.745477 | -2.087637 |
| 1 | 0 | 4.194778 | -2.176246 | -0.538432 |
| 1 | 0 | 0.492315 | -0.249400 | 1.926880 |
| 1 | 0 | -0.930319 | -0.381831 | 0.871286 |
| 1 | 0 | 2.522700 | -1.182102 | 0.989228 |
| 1 | 0 | -1.096106 | -2.855575 | -0.405769 |
| 1 | 0 | 1.035111 | -3.970288 | -1.817122 |
| 1 | 0 | 0.954008 | 0.763122 | -3.507669 |
| 8 | 0 | 1.278180 | 0.084090 | -1.559758 |
| 1 | 0 | -0.450823 | 0.156199 | -2.590389 |
| 1 | 0 | -1.442228 | -0.963583 | 3.258327 |
| 8 | 0 | -1.960196 | -2.593838 | 2.056131 |
| 1 | 0 | -0.296218 | -2.317125 | 3.169473 |
| 1 | 0 | 2.051799 | 0.642068 | -1.337432 |
| 1 | 0 | -2.663904 | -2.059752 | 1.637846 |

**trimer ca**

*** imOH_BF4_b3lyp_6-31+Gp_trimer_D3.g09, E(RB3LYP) =  -2533.58418824

| | | | | |
|---|---|---|---|---|
| 7 | 0 | -4.064985 | -0.332992 | 1.265784 |

| | | | | |
|---|---|---|---|---|
| 6 | 0 | -4.366573 | 0.087975 | -0.017134 |
| 6 | 0 | -4.046948 | 1.412308 | -0.093849 |
| 7 | 0 | -3.547021 | 1.777878 | 1.142664 |
| 6 | 0 | -3.561927 | 0.705278 | 1.944411 |
| 6 | 0 | -3.054134 | 3.110004 | 1.521251 |
| 6 | 0 | -4.252609 | -1.707117 | 1.766127 |
| 6 | 0 | -3.214995 | -2.093921 | 2.830115 |
| 9 | 0 | -1.200337 | -2.755119 | 0.086003 |
| 5 | 0 | -2.248965 | -3.423288 | -0.648734 |
| 9 | 0 | -2.988475 | -4.198180 | 0.246152 |
| 9 | 0 | -1.657500 | -4.185156 | -1.662266 |
| 9 | 0 | -3.072199 | -2.414932 | -1.213638 |
| 1 | 0 | -5.264795 | -1.795689 | 2.177426 |
| 1 | 0 | -4.169918 | -2.362936 | 0.900640 |
| 1 | 0 | -3.045794 | 3.738437 | 0.632533 |
| 1 | 0 | -3.713438 | 3.541050 | 2.278875 |
| 1 | 0 | -2.031650 | 3.035507 | 1.897062 |
| 1 | 0 | -3.194620 | 0.679716 | 2.956888 |
| 1 | 0 | -4.059579 | 2.100315 | -0.925503 |
| 1 | 0 | -4.720804 | -0.604723 | -0.763467 |
| 1 | 0 | -3.308542 | -3.177619 | 2.984089 |
| 8 | 0 | -1.897777 | -1.716386 | 2.485450 |
| 1 | 0 | -3.433877 | -1.602517 | 3.785725 |
| 1 | 0 | -1.628022 | -2.167531 | 1.654516 |
| 7 | 0 | 1.490873 | -1.824402 | -2.014285 |
| 6 | 0 | 1.902852 | -0.500537 | -2.015113 |
| 6 | 0 | 0.842193 | 0.241347 | -2.449100 |
| 7 | 0 | -0.200100 | -0.640459 | -2.689371 |
| 6 | 0 | 0.216195 | -1.881712 | -2.420506 |
| 6 | 0 | -1.536874 | -0.278767 | -3.172868 |
| 6 | 0 | 2.282186 | -2.980378 | -1.567080 |
| 6 | 0 | 2.111410 | -3.256104 | -0.068341 |
| 9 | 0 | -0.390233 | 3.189987 | -2.661729 |
| 5 | 0 | -1.471528 | 3.187293 | -1.776210 |
| 9 | 0 | -2.695580 | 3.045759 | -2.439037 |
| 9 | 0 | -1.315286 | 2.085618 | -0.872322 |
| 9 | 0 | -1.473989 | 4.378244 | -0.996579 |
| 9 | 0 | 4.770713 | -1.318589 | -0.506558 |
| 5 | 0 | 5.291716 | -0.053712 | -0.051899 |
| 9 | 0 | 6.558584 | 0.154176 | -0.542443 |
| 9 | 0 | 5.264380 | -0.068410 | 1.368223 |
| 9 | 0 | 4.384710 | 0.953274 | -0.501779 |
| 6 | 0 | 2.584576 | 1.050780 | 1.832497 |
| 7 | 0 | 1.602320 | 1.587662 | 1.099321 |
| 6 | 0 | 0.417752 | 0.934061 | 1.398354 |
| 6 | 0 | 0.711803 | -0.014583 | 2.333115 |
| 7 | 0 | 2.067941 | 0.080371 | 2.592515 |
| 6 | 0 | 1.770575 | 2.721726 | 0.180761 |
| 6 | 0 | 1.443265 | 4.055000 | 0.862378 |
| 6 | 0 | 2.837700 | -0.806854 | 3.466567 |
| 1 | 0 | 1.960241 | -3.851188 | -2.146188 |
| 1 | 0 | 3.325924 | -2.763694 | -1.804612 |
| 1 | 0 | -1.905102 | 0.559873 | -2.583587 |
| 1 | 0 | -1.478991 | 0.008696 | -4.225703 |
| 1 | 0 | -2.196099 | -1.136956 | -3.040327 |
| 1 | 0 | -0.383754 | -2.778318 | -2.481422 |
| 1 | 0 | 0.717762 | 1.301513 | -2.606186 |
| 1 | 0 | 2.892411 | -0.211067 | -1.691986 |
| 1 | 0 | 2.580287 | -0.608911 | 4.510622 |
| 1 | 0 | 2.605461 | -1.837173 | 3.194305 |
| 1 | 0 | 3.899110 | -0.627111 | 3.292318 |
| 1 | 0 | 2.803258 | 2.698717 | -0.177433 |
| 1 | 0 | 1.101295 | 2.559812 | -0.663768 |
| 1 | 0 | 3.631201 | 1.300292 | 1.766780 |

| | | | | |
|---|---|---|---|---|
| 1 | 0 | -0.496665 | 1.186975 | 0.889926 |
| 1 | 0 | 0.081046 | -0.757215 | 2.797012 |
| 1 | 0 | 2.701921 | -4.156385 | 0.166449 |
| 8 | 0 | 2.493878 | -2.156891 | 0.736933 |
| 1 | 0 | 1.061096 | -3.458498 | 0.155613 |
| 1 | 0 | 1.692191 | 4.863691 | 0.160144 |
| 8 | 0 | 0.091077 | 4.131250 | 1.284345 |
| 1 | 0 | 2.062804 | 4.179183 | 1.757502 |
| 1 | 0 | 3.401187 | -1.885907 | 0.485405 |
| 1 | 0 | -0.464044 | 4.286925 | 0.494585 |

**tetramer ca**

*** imOH_BF4_b3lyp_6-31+Gp_tetramer_D3.g09, E(RB3LYP) = -3378.11575625

| | | | | |
|---|---|---|---|---|
| 7 | 0 | 5.957772 | -0.794729 | 0.751501 |
| 6 | 0 | 6.212942 | -0.176621 | -0.460819 |
| 6 | 0 | 6.231346 | -1.159585 | -1.405661 |
| 7 | 0 | 5.980658 | -2.359129 | -0.758375 |
| 6 | 0 | 5.808439 | -2.109700 | 0.543591 |
| 6 | 0 | 5.933334 | -3.679231 | -1.394365 |
| 6 | 0 | 5.806651 | -0.104670 | 2.043320 |
| 6 | 0 | 4.393552 | 0.455038 | 2.256973 |
| 9 | 0 | 2.995800 | -3.162769 | -1.284385 |
| 5 | 0 | 2.731328 | -3.254876 | 0.128969 |
| 9 | 0 | 3.629475 | -4.168190 | 0.700410 |
| 9 | 0 | 2.934716 | -1.973312 | 0.683700 |
| 9 | 0 | 1.399025 | -3.652173 | 0.297071 |
| 9 | 0 | 6.011063 | 2.929302 | 0.357727 |
| 5 | 0 | 5.573146 | 3.560442 | -0.870368 |
| 9 | 0 | 6.603844 | 4.285997 | -1.421564 |
| 9 | 0 | 4.448088 | 4.361730 | -0.569648 |
| 9 | 0 | 5.154025 | 2.509032 | -1.741601 |
| 6 | 0 | 2.195904 | 2.375681 | -0.765900 |
| 7 | 0 | 2.019899 | 1.053219 | -0.858265 |
| 6 | 0 | 1.164873 | 0.643575 | 0.151059 |
| 6 | 0 | 0.807610 | 1.761046 | 0.844937 |
| 7 | 0 | 1.463270 | 2.829155 | 0.256791 |
| 6 | 0 | 2.764845 | 0.191708 | -1.780486 |
| 6 | 0 | 1.861516 | -0.504667 | -2.798164 |
| 6 | 0 | 1.437300 | 4.216333 | 0.720727 |
| 1 | 0 | 6.058364 | -0.816470 | 2.835329 |
| 1 | 0 | 6.546269 | 0.701064 | 2.054315 |
| 1 | 0 | 5.249396 | -3.635409 | -2.242138 |
| 1 | 0 | 6.938919 | -3.960159 | -1.718435 |
| 1 | 0 | 5.540366 | -4.398710 | -0.677586 |
| 1 | 0 | 5.531266 | -2.844271 | 1.283108 |
| 1 | 0 | 6.397214 | -1.110398 | -2.470455 |
| 1 | 0 | 6.325500 | 0.893209 | -0.543056 |
| 1 | 0 | 0.512065 | 4.703098 | 0.399334 |
| 1 | 0 | 1.494438 | 4.220243 | 1.811226 |
| 1 | 0 | 2.304819 | 4.737044 | 0.313310 |
| 1 | 0 | 3.503369 | 0.824042 | -2.279433 |
| 1 | 0 | 3.295352 | -0.545290 | -1.176317 |
| 1 | 0 | 2.881401 | 2.963737 | -1.355126 |
| 1 | 0 | 0.924277 | -0.390555 | 0.319746 |
| 1 | 0 | 0.165661 | 1.875960 | 1.704652 |
| 1 | 0 | 4.388301 | 0.945372 | 3.244247 |
| 8 | 0 | 3.998569 | 1.333134 | 1.228389 |
| 1 | 0 | 3.662541 | -0.357145 | 2.268188 |
| 1 | 0 | 2.500135 | -0.950925 | -3.574803 |
| 8 | 0 | 1.022136 | -1.489318 | -2.207510 |
| 1 | 0 | 1.201051 | 0.223079 | -3.281994 |

| | | | | |
|---|---|---|---|---|
| 1 | 0 | 4.676094 | 2.025410 | 1.081383 |
| 1 | 0 | 1.595602 | -2.231450 | -1.929223 |
| 7 | 0 | -2.416142 | -2.000209 | -0.997043 |
| 6 | 0 | -3.653124 | -2.274386 | -0.442454 |
| 6 | 0 | -3.417455 | -2.939246 | 0.725498 |
| 7 | 0 | -2.045656 | -3.056814 | 0.865758 |
| 6 | 0 | -1.461204 | -2.473673 | -0.184641 |
| 6 | 0 | -1.353946 | -3.664740 | 2.007707 |
| 6 | 0 | -2.174625 | -1.245452 | -2.235896 |
| 6 | 0 | -2.185493 | 0.270841 | -2.013007 |
| 9 | 0 | -2.394627 | -0.875434 | 3.353879 |
| 5 | 0 | -1.441950 | -0.140280 | 2.581980 |
| 9 | 0 | -0.220073 | -0.818342 | 2.556099 |
| 9 | 0 | -1.936387 | -0.011049 | 1.262727 |
| 9 | 0 | -1.302800 | 1.147730 | 3.142437 |
| 9 | 0 | -5.288562 | -0.734299 | -2.954985 |
| 5 | 0 | -6.611486 | -0.597688 | -2.398083 |
| 9 | 0 | -7.508266 | -1.382181 | -3.082883 |
| 9 | 0 | -6.956096 | 0.777534 | -2.441191 |
| 9 | 0 | -6.520457 | -0.983903 | -1.023836 |
| 6 | 0 | -5.603862 | 1.747310 | 0.082294 |
| 7 | 0 | -4.908051 | 1.363722 | 1.156968 |
| 6 | 0 | -3.918727 | 2.298848 | 1.408340 |
| 6 | 0 | -4.048651 | 3.268021 | 0.458099 |
| 7 | 0 | -5.108407 | 2.907236 | -0.357491 |
| 6 | 0 | -5.111632 | 0.089842 | 1.861187 |
| 6 | 0 | -5.478102 | 0.280469 | 3.334501 |
| 6 | 0 | -5.511978 | 3.576763 | -1.593100 |
| 1 | 0 | -1.198096 | -1.550269 | -2.616820 |
| 1 | 0 | -2.952929 | -1.535606 | -2.946290 |
| 1 | 0 | -1.580441 | -3.082118 | 2.901518 |
| 1 | 0 | -1.693666 | -4.697896 | 2.117312 |
| 1 | 0 | -0.282486 | -3.642988 | 1.817360 |
| 1 | 0 | -0.397913 | -2.405934 | -0.349992 |
| 1 | 0 | -4.096841 | -3.326942 | 1.468083 |
| 1 | 0 | -4.573660 | -1.959560 | -0.911536 |
| 1 | 0 | -5.841164 | 4.595361 | -1.371366 |
| 1 | 0 | -4.659671 | 3.593619 | -2.276998 |
| 1 | 0 | -6.320028 | 3.006151 | -2.051458 |
| 1 | 0 | -5.901779 | -0.444846 | 1.326911 |
| 1 | 0 | -4.184841 | -0.475481 | 1.766190 |
| 1 | 0 | -6.392366 | 1.184857 | -0.392391 |
| 1 | 0 | -3.221461 | 2.179068 | 2.220854 |
| 1 | 0 | -3.481200 | 4.168758 | 0.284323 |
| 1 | 0 | -1.837175 | 0.732334 | -2.952944 |
| 8 | 0 | -3.448593 | 0.782721 | -1.629494 |
| 1 | 0 | -1.485350 | 0.534871 | -1.215303 |
| 1 | 0 | -5.702633 | -0.715209 | 3.749096 |
| 8 | 0 | -4.471281 | 0.943035 | 4.076866 |
| 1 | 0 | -6.381428 | 0.893415 | 3.424923 |
| 1 | 0 | -4.137572 | 0.392802 | -2.205997 |
| 1 | 0 | -3.670905 | 0.384335 | 4.082781 |

## dimer cc

*** imOH_BF4_cation_cation_b3lyp_6-31+Gp_dimer_D3.g09, E(RB3LYP) = -1689.03077609

| | | | | |
|---|---|---|---|---|
| 6 | 0 | 0.661378 | -3.831511 | -0.936789 |
| 6 | 0 | -0.205805 | -3.091176 | -1.682808 |
| 7 | 0 | -0.599404 | -2.021456 | -0.898186 |
| 6 | 0 | 0.012666 | -2.107397 | 0.286650 |
| 7 | 0 | 0.777986 | -3.201704 | 0.290184 |

| | | | | |
|---|---|---|---|---|
| 6 | 0 | -1.402857 | -0.871893 | -1.318061 |
| 6 | 0 | -2.899534 | -1.169905 | -1.497565 |
| 8 | 0 | -3.611299 | 0.067730 | -1.497458 |
| 6 | 0 | 1.737561 | -3.544597 | 1.340480 |
| 9 | 0 | -2.839695 | 0.841067 | 1.062326 |
| 5 | 0 | -2.476290 | -0.137043 | 2.077163 |
| 9 | 0 | -2.730395 | -1.414351 | 1.540804 |
| 9 | 0 | -1.072920 | -0.007463 | 2.281446 |
| 9 | 0 | -3.179143 | 0.105535 | 3.234614 |
| 8 | 0 | -2.410707 | 2.397951 | -2.669753 |
| 6 | 0 | -1.737183 | 2.837716 | -1.503548 |
| 6 | 0 | -0.242950 | 2.502440 | -1.646063 |
| 7 | 0 | 0.515219 | 2.595114 | -0.380891 |
| 6 | 0 | 0.038845 | 2.335858 | 0.894814 |
| 6 | 0 | 1.120946 | 2.281362 | 1.720145 |
| 7 | 0 | 2.241439 | 2.502569 | 0.942785 |
| 6 | 0 | 1.851693 | 2.672853 | -0.323621 |
| 6 | 0 | 3.626221 | 2.401864 | 1.406396 |
| 9 | 0 | 3.749402 | 0.456090 | -0.861953 |
| 5 | 0 | 2.726031 | -0.475675 | -0.599073 |
| 9 | 0 | 3.091450 | -1.770955 | -0.985665 |
| 9 | 0 | 1.550981 | -0.075589 | -1.300465 |
| 9 | 0 | 2.433450 | -0.468357 | 0.795768 |
| 1 | 0 | -0.556121 | -3.215655 | -2.695321 |
| 1 | 0 | -0.088428 | -1.394123 | 1.092866 |
| 1 | 0 | -1.288622 | -0.119819 | -0.539434 |
| 1 | 0 | -0.960461 | -0.483636 | -2.237975 |
| 1 | 0 | -3.099882 | -1.648712 | -2.461188 |
| 1 | 0 | -3.249670 | -1.819912 | -0.690694 |
| 1 | 0 | -3.646381 | 0.369847 | -0.566104 |
| 1 | 0 | -2.895647 | 1.575752 | -2.442917 |
| 1 | 0 | -1.874158 | 3.921271 | -1.401404 |
| 1 | 0 | -2.157982 | 2.361633 | -0.613152 |
| 1 | 0 | -0.123323 | 1.481773 | -2.008420 |
| 1 | 0 | 0.229215 | 3.173466 | -2.368465 |
| 1 | 0 | 2.513030 | 2.761981 | -1.170144 |
| 1 | 0 | -1.001574 | 2.165066 | 1.113224 |
| 1 | 0 | 1.184359 | 2.067026 | 2.774891 |
| 1 | 0 | 3.823594 | 1.361792 | 1.670007 |
| 1 | 0 | 4.293579 | 2.690788 | 0.595248 |
| 1 | 0 | 3.765482 | 3.063539 | 2.264576 |
| 1 | 0 | 1.714978 | -4.623485 | 1.508921 |
| 1 | 0 | 2.728472 | -3.214482 | 1.022107 |
| 1 | 0 | 1.454899 | -3.022963 | 2.255356 |
| 1 | 0 | 1.221250 | -4.722133 | -1.174027 |

## trimer cc

*** imOH_BF4_cation_cation_b3lyp_6-31+Gp_trimer_D3.g09, E(RB3LYP) = -2533.55624132

| | | | | |
|---|---|---|---|---|
| 6 | 0 | -5.585765 | 0.961949 | -0.302834 |
| 7 | 0 | -4.689276 | -0.028361 | -0.194928 |
| 6 | 0 | -4.573977 | -0.383411 | 1.137490 |
| 6 | 0 | -5.436725 | 0.411900 | 1.831719 |
| 7 | 0 | -6.061912 | 1.239849 | 0.914199 |
| 6 | 0 | -3.868026 | -0.582323 | -1.284923 |
| 6 | 0 | -2.772240 | 0.398536 | -1.729217 |
| 8 | 0 | -2.046564 | 0.902319 | -0.613377 |
| 6 | 0 | -7.008587 | 2.307810 | 1.242909 |
| 9 | 0 | -2.195610 | -2.565982 | 1.384621 |
| 5 | 0 | -2.805441 | -3.640369 | 0.661759 |
| 9 | 0 | -2.498236 | -3.462043 | -0.716573 |

| | | | | |
|---|---|---|---|---|
| 9 | 0 | -4.184654 | -3.599599 | 0.854284 |
| 9 | 0 | -2.238370 | -4.854902 | 1.102979 |
| 8 | 0 | 0.477507 | 0.471150 | 0.193863 |
| 6 | 0 | 0.240388 | -0.360835 | 1.330083 |
| 6 | 0 | 1.242686 | -1.511106 | 1.338681 |
| 7 | 0 | 1.053149 | -2.411625 | 0.193557 |
| 6 | 0 | 0.388980 | -3.572932 | 0.223134 |
| 7 | 0 | 0.377482 | -4.089881 | -1.008130 |
| 6 | 0 | 1.050446 | -3.223150 | -1.854391 |
| 6 | 0 | 1.476132 | -2.169621 | -1.100493 |
| 6 | 0 | -0.296665 | -5.326588 | -1.406957 |
| 8 | 0 | 1.738428 | 2.696851 | 1.178978 |
| 6 | 0 | 3.104073 | 2.368695 | 1.240799 |
| 6 | 0 | 3.818050 | 2.926679 | -0.006416 |
| 7 | 0 | 5.272088 | 2.691574 | 0.063499 |
| 6 | 0 | 6.129367 | 3.224898 | 1.010529 |
| 6 | 0 | 7.341583 | 2.623624 | 0.837814 |
| 7 | 0 | 7.206643 | 1.735300 | -0.214178 |
| 6 | 0 | 5.946207 | 1.791189 | -0.657086 |
| 6 | 0 | 8.206761 | 0.753336 | -0.644293 |
| 9 | 0 | 4.217561 | -2.241659 | 0.253760 |
| 5 | 0 | 4.798364 | -1.015419 | -0.057326 |
| 9 | 0 | 3.840524 | -0.194998 | -0.751187 |
| 9 | 0 | 5.926049 | -1.163886 | -0.897095 |
| 9 | 0 | 5.183127 | -0.322749 | 1.115214 |
| 9 | 0 | -3.474434 | 4.967013 | -1.197835 |
| 5 | 0 | -4.239479 | 3.857378 | -0.908904 |
| 9 | 0 | -3.541608 | 3.011624 | 0.047853 |
| 9 | 0 | -5.483761 | 4.187443 | -0.340433 |
| 9 | 0 | -4.465063 | 3.063787 | -2.070301 |
| 1 | 0 | -2.480930 | 1.735025 | -0.315988 |
| 1 | 0 | -4.524660 | -0.827649 | -2.125952 |
| 1 | 0 | -3.435912 | -1.508746 | -0.911060 |
| 1 | 0 | -2.073794 | -0.151719 | -2.369467 |
| 1 | 0 | -3.198399 | 1.229382 | -2.298449 |
| 1 | 0 | -0.399830 | 0.687367 | -0.208450 |
| 1 | 0 | -0.781461 | -0.749966 | 1.311912 |
| 1 | 0 | 0.382770 | 0.216767 | 2.255174 |
| 1 | 0 | 2.270379 | -1.147780 | 1.291335 |
| 1 | 0 | 1.124268 | -2.104925 | 2.248905 |
| 1 | 0 | 1.254884 | 1.962465 | 0.730988 |
| 1 | 0 | 3.287225 | 1.289903 | 1.299182 |
| 1 | 0 | 3.523615 | 2.834394 | 2.142444 |
| 1 | 0 | 3.641503 | 4.001937 | -0.095788 |
| 1 | 0 | 3.446499 | 2.424796 | -0.900991 |
| 1 | 0 | -0.113788 | -3.995302 | 1.078811 |
| 1 | 0 | 1.175862 | -3.439979 | -2.903560 |
| 1 | 0 | 2.066414 | -1.299672 | -1.336397 |
| 1 | 0 | 5.805492 | 3.978420 | 1.711049 |
| 1 | 0 | 8.276254 | 2.747123 | 1.362333 |
| 1 | 0 | 5.523026 | 1.161731 | -1.422613 |
| 1 | 0 | -3.912380 | -1.175904 | 1.453498 |
| 1 | 0 | -5.665067 | 0.458245 | 2.884890 |
| 1 | 0 | -5.816903 | 1.505565 | -1.206082 |
| 1 | 0 | 7.985128 | 0.443745 | -1.665009 |
| 1 | 0 | 9.195136 | 1.214893 | -0.598128 |
| 1 | 0 | 8.147227 | -0.121914 | 0.005231 |
| 1 | 0 | -7.451180 | 2.685659 | 0.322764 |
| 1 | 0 | -7.781934 | 1.902967 | 1.900181 |
| 1 | 0 | -6.471665 | 3.128057 | 1.721498 |
| 1 | 0 | -1.037351 | -5.091462 | -2.172951 |
| 1 | 0 | 0.443557 | -6.036670 | -1.785369 |
| 1 | 0 | -0.819230 | -5.733602 | -0.542335 |

**tetramer cc**

*** imOH_BF4_cation_cation_b3lyp_6-31+Gp_tetramer_l_D3.g09, E(RB3LYP) = -3378.10686835

| | | | | |
|---|---|---|---|---|
| 6 | 0 | 3.375127 | 4.822319 | -0.243667 |
| 7 | 0 | 3.238869 | 3.479826 | 0.071855 |
| 6 | 0 | 4.434876 | 2.991169 | 0.420134 |
| 7 | 0 | 5.337828 | 3.975509 | 0.342540 |
| 6 | 0 | 4.692573 | 5.131395 | -0.069533 |
| 6 | 0 | 2.021850 | 2.664146 | -0.086616 |
| 6 | 0 | 1.744314 | 2.365880 | -1.571063 |
| 8 | 0 | 0.722161 | 1.384699 | -1.671094 |
| 6 | 0 | 6.763847 | 3.840066 | 0.647752 |
| 8 | 0 | 1.463272 | -1.225182 | -1.576939 |
| 6 | 0 | 2.237157 | -1.797734 | -0.531249 |
| 6 | 0 | 3.174882 | -2.865097 | -1.119108 |
| 7 | 0 | 3.772144 | -3.672863 | -0.039755 |
| 6 | 0 | 4.665224 | -3.229202 | 0.922698 |
| 6 | 0 | 4.843997 | -4.262017 | 1.795925 |
| 7 | 0 | 4.056401 | -5.314878 | 1.357730 |
| 6 | 0 | 3.418752 | -4.930807 | 0.246307 |
| 6 | 0 | 3.924803 | -6.624071 | 2.002301 |
| 8 | 0 | -1.862225 | 1.235709 | -0.757099 |
| 6 | 0 | -2.562653 | 2.150290 | -1.586661 |
| 6 | 0 | -3.844648 | 2.573603 | -0.877912 |
| 7 | 0 | -3.556742 | 3.169312 | 0.439494 |
| 6 | 0 | -2.989819 | 4.365089 | 0.640958 |
| 7 | 0 | -2.806160 | 4.533094 | 1.956153 |
| 6 | 0 | -3.264271 | 3.400903 | 2.611122 |
| 6 | 0 | -3.732687 | 2.543995 | 1.658496 |
| 6 | 0 | -2.197713 | 5.702780 | 2.596300 |
| 9 | 0 | -5.243987 | -0.208981 | 1.341537 |
| 5 | 0 | -6.365466 | 0.184626 | 0.517210 |
| 9 | 0 | -7.426001 | -0.696671 | 0.761430 |
| 9 | 0 | -5.943862 | 0.036349 | -0.835728 |
| 9 | 0 | -6.680369 | 1.511262 | 0.786897 |
| 8 | 0 | -0.834182 | -2.629955 | -1.115252 |
| 6 | 0 | -1.379374 | -2.026639 | 0.059726 |
| 6 | 0 | -2.389172 | -2.928202 | 0.772669 |
| 7 | 0 | -3.482600 | -3.342075 | -0.118642 |
| 6 | 0 | -4.639212 | -2.682293 | -0.258486 |
| 7 | 0 | -5.367651 | -3.297165 | -1.195954 |
| 6 | 0 | -4.646004 | -4.380147 | -1.675072 |
| 6 | 0 | -3.460938 | -4.409245 | -0.999536 |
| 6 | 0 | -6.694308 | -2.869433 | -1.658404 |
| 9 | 0 | 4.403878 | 0.489103 | -1.422278 |
| 5 | 0 | 5.482836 | 0.035874 | -0.637864 |
| 9 | 0 | 5.903417 | -1.232860 | -1.056004 |
| 9 | 0 | 5.004788 | -0.064141 | 0.722927 |
| 9 | 0 | 6.532393 | 0.967222 | -0.662982 |
| 9 | 0 | -1.167287 | -5.933750 | 0.507735 |
| 5 | 0 | 0.188337 | -5.592213 | 0.482390 |
| 9 | 0 | 0.475221 | -4.614106 | 1.454756 |
| 9 | 0 | 0.484238 | -4.998333 | -0.805161 |
| 9 | 0 | 1.013089 | -6.714251 | 0.646754 |
| 9 | 0 | -0.463124 | 4.694162 | -1.223192 |
| 5 | 0 | -0.111110 | 5.817368 | -0.436924 |
| 9 | 0 | -1.242087 | 6.651500 | -0.286217 |
| 9 | 0 | 0.301645 | 5.348161 | 0.841915 |
| 9 | 0 | 0.945434 | 6.516307 | -1.042148 |
| 1 | 0 | -0.910925 | 1.299939 | -1.000090 |

| | | | | |
|---|---|---|---|---|
| 1 | 0 | -4.392572 | 3.308622 | -1.475089 |
| 1 | 0 | -4.499263 | 1.721103 | -0.706536 |
| 1 | 0 | -2.854120 | 1.684955 | -2.541689 |
| 1 | 0 | -1.941040 | 3.026444 | -1.804636 |
| 1 | 0 | -0.411277 | -3.490507 | -0.908208 |
| 1 | 0 | -1.842628 | -1.091166 | -0.266385 |
| 1 | 0 | -0.590207 | -1.786258 | 0.786824 |
| 1 | 0 | -1.914681 | -3.834915 | 1.150156 |
| 1 | 0 | -2.832788 | -2.386020 | 1.612722 |
| 1 | 0 | 0.597330 | -1.699246 | -1.611037 |
| 1 | 0 | 1.595177 | -2.261828 | 0.228679 |
| 1 | 0 | 2.825694 | -1.003567 | -0.065668 |
| 1 | 0 | 3.978651 | -2.403294 | -1.693467 |
| 1 | 0 | 2.604075 | -3.546672 | -1.752976 |
| 1 | 0 | 1.112663 | 0.477814 | -1.652841 |
| 1 | 0 | 1.382899 | 3.270282 | -2.066639 |
| 1 | 0 | 2.663178 | 2.016776 | -2.054665 |
| 1 | 0 | 2.191760 | 1.728514 | 0.451899 |
| 1 | 0 | 1.188132 | 3.205010 | 0.364544 |
| 1 | 0 | -4.920883 | -1.799607 | 0.299402 |
| 1 | 0 | -5.041295 | -5.031401 | -2.438955 |
| 1 | 0 | -2.626615 | -5.092318 | -1.034070 |
| 1 | 0 | 5.081915 | -2.234454 | 0.891213 |
| 1 | 0 | 5.455599 | -4.340756 | 2.681232 |
| 1 | 0 | 2.708001 | -5.525151 | -0.304284 |
| 1 | 0 | 4.639189 | 1.967957 | 0.697472 |
| 1 | 0 | 5.221557 | 6.062475 | -0.201070 |
| 1 | 0 | 2.535320 | 5.432455 | -0.547631 |
| 1 | 0 | -4.193605 | 1.570170 | 1.729979 |
| 1 | 0 | -3.227288 | 3.313952 | 3.685724 |
| 1 | 0 | -2.689044 | 5.069624 | -0.119531 |
| 1 | 0 | 3.033480 | -7.115286 | 1.612117 |
| 1 | 0 | 4.816168 | -7.225893 | 1.805042 |
| 1 | 0 | 3.807365 | -6.474784 | 3.077793 |
| 1 | 0 | 7.336937 | 4.376861 | -0.111233 |
| 1 | 0 | 6.974201 | 4.258225 | 1.636166 |
| 1 | 0 | 7.028201 | 2.783295 | 0.607787 |
| 1 | 0 | -2.014027 | 6.462403 | 1.838544 |
| 1 | 0 | -2.877297 | 6.079247 | 3.365173 |
| 1 | 0 | -1.240068 | 5.414933 | 3.034399 |
| 1 | 0 | -6.591922 | -2.328296 | -2.601864 |
| 1 | 0 | -7.316658 | -3.756604 | -1.794650 |
| 1 | 0 | -7.135195 | -2.208026 | -0.913261 |

**tetramer cc_cyc**

*** imOH_BF4_cation_cation_b3lyp_6-31+Gp_tetramer_D3.g09, E(RB3LYP) = -3378.11423979

| | | | | |
|---|---|---|---|---|
| 6 | 0 | -3.142778 | 3.321540 | 0.143202 |
| 7 | 0 | -3.977421 | 2.392172 | -0.341968 |
| 6 | 0 | -5.279505 | 2.758963 | -0.042073 |
| 6 | 0 | -5.210077 | 3.942325 | 0.629066 |
| 7 | 0 | -3.869314 | 4.276034 | 0.734239 |
| 6 | 0 | -3.605389 | 1.158480 | -1.064034 |
| 6 | 0 | -2.164001 | 1.160890 | -1.535842 |
| 8 | 0 | -1.318939 | 1.095798 | -0.387532 |
| 6 | 0 | -3.335193 | 5.506473 | 1.324291 |
| 9 | 0 | -5.101429 | -0.397462 | 1.344496 |
| 5 | 0 | -6.420761 | -0.381544 | 0.798506 |
| 9 | 0 | -6.296830 | -0.196792 | -0.612844 |
| 9 | 0 | -7.162840 | 0.658950 | 1.345480 |
| 9 | 0 | -7.021599 | -1.639275 | 1.042938 |

| | | | | |
|---|---|---|---|---|
| 8 | 0 | -1.028569 | -1.290581 | 0.812209 |
| 6 | 0 | -1.966099 | -1.594068 | 1.842243 |
| 6 | 0 | -2.497342 | -3.010062 | 1.629732 |
| 7 | 0 | -3.205342 | -3.122665 | 0.345247 |
| 6 | 0 | -4.531608 | -3.034450 | 0.184676 |
| 7 | 0 | -4.808850 | -3.091411 | -1.121603 |
| 6 | 0 | -3.615291 | -3.216597 | -1.818219 |
| 6 | 0 | -2.607970 | -3.236545 | -0.898544 |
| 6 | 0 | -6.146903 | -3.018139 | -1.714851 |
| 8 | 0 | 1.607958 | -0.777006 | 1.139046 |
| 6 | 0 | 2.577351 | -1.821023 | 1.087844 |
| 6 | 0 | 2.925462 | -2.120166 | -0.380475 |
| 7 | 0 | 3.810732 | -3.293920 | -0.465338 |
| 6 | 0 | 5.154845 | -3.340761 | -0.129711 |
| 6 | 0 | 5.528301 | -4.652067 | -0.204073 |
| 7 | 0 | 4.406910 | -5.379869 | -0.575500 |
| 6 | 0 | 3.384071 | -4.532418 | -0.730652 |
| 6 | 0 | 4.318724 | -6.830309 | -0.758043 |
| 8 | 0 | 1.284684 | 0.929757 | -0.931183 |
| 6 | 0 | 2.237139 | 1.985644 | -0.962994 |
| 6 | 0 | 2.202346 | 2.763020 | 0.362723 |
| 7 | 0 | 3.256739 | 3.791375 | 0.366544 |
| 6 | 0 | 4.511694 | 3.569074 | 0.772294 |
| 7 | 0 | 5.251537 | 4.644239 | 0.479934 |
| 6 | 0 | 4.436369 | 5.587071 | -0.129659 |
| 6 | 0 | 3.183490 | 5.050205 | -0.208949 |
| 6 | 0 | 6.685802 | 4.766210 | 0.750664 |
| 9 | 0 | 4.693476 | 0.630545 | 1.185499 |
| 5 | 0 | 5.791053 | 0.363612 | 0.308287 |
| 9 | 0 | 6.515066 | -0.738914 | 0.793708 |
| 9 | 0 | 6.608365 | 1.515204 | 0.258445 |
| 9 | 0 | 5.271491 | 0.072713 | -0.969931 |
| 9 | 0 | -1.109344 | -5.620537 | 0.321529 |
| 5 | 0 | 0.222769 | -5.212707 | 0.227745 |
| 9 | 0 | 0.372742 | -4.339720 | -0.909777 |
| 9 | 0 | 1.091681 | -6.311013 | 0.043207 |
| 9 | 0 | 0.598549 | -4.485010 | 1.377325 |
| 9 | 0 | 0.517250 | 5.918122 | -1.522721 |
| 5 | 0 | -0.469118 | 5.034518 | -1.058857 |
| 9 | 0 | -0.310467 | 4.859878 | 0.357701 |
| 9 | 0 | -1.759475 | 5.520516 | -1.310232 |
| 9 | 0 | -0.304506 | 3.760543 | -1.657534 |
| 1 | 0 | -0.370128 | 1.216430 | -0.664071 |
| 1 | 0 | -4.285052 | 1.071688 | -1.914837 |
| 1 | 0 | -3.802647 | 0.319457 | -0.394580 |
| 1 | 0 | -2.019531 | 0.277901 | -2.175171 |
| 1 | 0 | -1.930423 | 2.059131 | -2.120014 |
| 1 | 0 | -1.233897 | -0.410395 | 0.404377 |
| 1 | 0 | -2.793020 | -0.875120 | 1.840811 |
| 1 | 0 | -1.471374 | -1.559929 | 2.822535 |
| 1 | 0 | -1.682924 | -3.735169 | 1.619023 |
| 1 | 0 | -3.201747 | -3.276078 | 2.422637 |
| 1 | 0 | 0.692755 | -1.151703 | 1.102988 |
| 1 | 0 | 2.191904 | -2.727599 | 1.566415 |
| 1 | 0 | 3.463903 | -1.463803 | 1.617671 |
| 1 | 0 | 3.438419 | -1.275960 | -0.843898 |
| 1 | 0 | 2.016076 | -2.359697 | -0.935756 |
| 1 | 0 | 1.522374 | 0.297935 | -0.203655 |
| 1 | 0 | 1.954299 | 2.641908 | -1.789135 |
| 1 | 0 | 3.244585 | 1.584989 | -1.134224 |
| 1 | 0 | 2.398261 | 2.086341 | 1.198043 |
| 1 | 0 | 1.242426 | 3.259992 | 0.503358 |
| 1 | 0 | -5.261205 | -2.883717 | 0.965012 |
| 1 | 0 | -3.592933 | -3.300971 | -2.893516 |

| | | | | |
|---|---|---|---|---|
| 1 | 0 | -1.541271 | -3.355422 | -1.004650 |
| 1 | 0 | 5.710189 | -2.448862 | 0.131894 |
| 1 | 0 | 6.481241 | -5.125598 | -0.025656 |
| 1 | 0 | 2.373280 | -4.799164 | -0.995750 |
| 1 | 0 | 4.865836 | 2.649314 | 1.212841 |
| 1 | 0 | 4.815798 | 6.544033 | -0.452746 |
| 1 | 0 | 2.260910 | 5.437868 | -0.622587 |
| 1 | 0 | -6.116780 | 2.126358 | -0.290173 |
| 1 | 0 | -5.986633 | 4.561870 | 1.049414 |
| 1 | 0 | -2.068813 | 3.304079 | 0.072595 |
| 1 | 0 | 3.266326 | -7.117611 | -0.723378 |
| 1 | 0 | 4.762515 | -7.115189 | -1.716013 |
| 1 | 0 | 4.852554 | -7.322133 | 0.058063 |
| 1 | 0 | 7.165292 | 5.246855 | -0.104705 |
| 1 | 0 | 6.848340 | 5.364989 | 1.651174 |
| 1 | 0 | 7.097712 | 3.763542 | 0.875594 |
| 1 | 0 | -2.250704 | 5.426908 | 1.381136 |
| 1 | 0 | -3.589545 | 6.352081 | 0.681567 |
| 1 | 0 | -3.765828 | 5.636677 | 2.320166 |
| 1 | 0 | -6.197294 | -2.149393 | -2.373125 |
| 1 | 0 | -6.344126 | -3.938874 | -2.270167 |
| 1 | 0 | -6.874655 | -2.882261 | -0.916381 |

## SI5 DFT-D3 calculated frequencies of the [HEMim][$BF_4$] clusters ca and cc

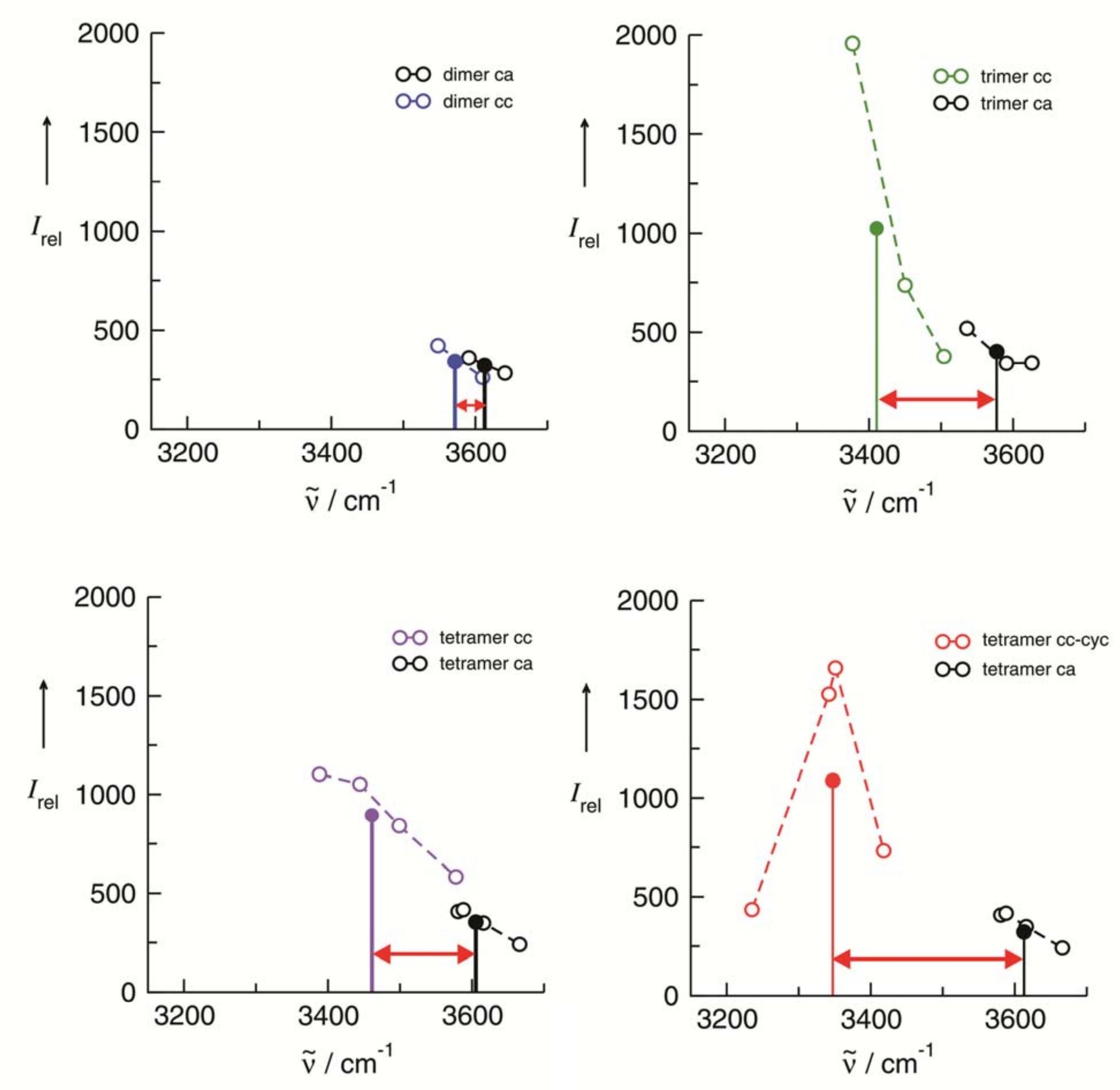


**SI-FIG3** DFT-D3 calculated frequencies and intensities of the OH stretches in the dimer cc (blue), the trimer cc (green), the tetramer cc (purple) and the tetramer cc-cyc (red) compared to the corresponding values of the ca clusters n=2-4 (black). The average values for each cluster ca and cc are given by the filled symbols.

## SI6 DFT-D3 calculated energies, free energies and NBO analysis of $[HEMim][BF_4]$ clusters ca and cc

***SI Table 1.*** Total energies (in Hartrees) for clusters **ca**.

| | Dimer ca | Trimer ca | Tetramer ca |
|---|---|---|---|
| **E** | 1689.040276 | -2533.584188 | -3378.115756 |
| **G** | -1688.722107 | -2533.094097 | -3377.458682 |

***SI Table 2.*** Total energies (in Hartrees) for clusters **cc**.

| | Dimer cc | Trimer cc | Tetramer cc | Tetramer cc-cyc |
|---|---|---|---|---|
| **E** | -1689.030776 | -2533.556241 | -3378.106868 | -3378.114239 |
| **G** | -1688.713597 | -2533.075443 | -3377.454176 | -3377.458989 |

***SI Table 3.*** Energy and free energy differences **ca-cc** per ion-pair (in kJ $mol^{-1}$).

| | Dimer cc | Trimer cc | Tetramer cc | Tetramer cc-cyc |
|---|---|---|---|---|
| **ΔE** | +12.47 | +24.46 | +5.83 | +1.0 |
| **ΔG** | +11.17 | +16.32 | +2.96 | +0.2 |

**NBO Analysis**

For the ion pairs the wavefunctions were analyzed by the natural bond orbital (NBO) method.[14,32] NBO analysis transforms the delocalized many-electron wavefunctions into optimized electron-pair bonding units, corresponding to the Lewis structure picture. Starting from a given input atomic orbital basis set, the program performs a transformation to form a set of high-occupancy Lewis-type (bond, lone pair) NBO's, each of which is taken to be doubly occupied. This represents the "natural Lewis structure" of the molecule. Delocalization effects, which appear as weak departures from the idealized localized picture, are reflected as nonzero occupancies of non-Lewis (antibond or Rydberg) NBO's. The total non-Lewis occupancy (1NL) constitutes a quantitative measure of electronic delocalization.The results of NBO analysis allow many of the quantitative trends in ion-pair structures, stability, and spectroscopic properties to be rationalized in terms of charge-transfer delocalization between ions or molecules.

***SI Table 4.*** NBO-Analysis of the IL clusters n=1-4.

```
      Donor NBO (i)  /Acceptor NBO (j)           kcal/mol   a.u.   a.u.
==============================================================================

dimer ca
========

  82. LP (1) F   9   /576. BD*(1) O  47 - H  50    2.63    1.37    0.054
  84. LP (3) F   9   /576. BD*(1) O  47 - H  50   10.00    0.89    0.085

  94. LP (1) F  14   /575. BD*(1) O  44 - H  49    2.37    1.34    0.051
  96. LP (3) F  14   /575. BD*(1) O  44 - H  49   14.27    0.90    0.102


trimer ca
=========

122. LP (1) F   9    /812. BD*(1) O  23 - H  25     3.81    1.34    0.064
124. LP (3) F   9    /812. BD*(1) O  23 - H  25    17.48    0.92    0.114

146. LP (1) F  38    /864. BD*(1) O  72 - H  75     3.09    1.35    0.058
148. LP (3) F  38    /864. BD*(1) O  72 - H  75    13.13    0.90    0.098

149. LP (1) F  39    /863. BD*(1) O  69 - H  74     2.63    1.34    0.053
151. LP (3) F  39    /863. BD*(1) O  69 - H  74    14.25    0.91    0.102


tetramer ca
===========

162. LP (1) F   9    /***. BD*(1) O  47 - H  50     2.47    1.37    0.052
164. LP (3) F   9    /***. BD*(1) O  47 - H  50    12.83    0.89    0.096

174. LP (1) F  14    /***. BD*(1) O  44 - H  49     2.65    1.33    0.053
176. LP (3) F  14    /***. BD*(1) O  44 - H  49    15.36    0.91    0.106

192. LP (1) F  59    /***. BD*(1) O  97 - H 100     2.07    1.39    0.048
194. LP (3) F  59    /***. BD*(1) O  97 - H 100     7.43    0.86    0.072

204. LP (1) F  64    /***. BD*(1) O  94 - H  99     2.68    1.34    0.054
```

```
206. LP (3) F  64    /***. BD*(1) O  94 - H  99    14.82    0.91    0.104


dimer cc
========

  82. LP (1) O   8   /552. BD*(1) O  15 - H  36    3.31    1.02    0.052
  83. LP (2) O   8   /552. BD*(1) O  15 - H  36   10.85    0.83    0.085

  84. LP (1) F  10   /543. BD*(1) O   8 - H  35    2.97    1.31    0.056
  86. LP (3) F  10   /543. BD*(1) O   8 - H  35    8.81    0.85    0.078


trimer cc
=========

122. LP (1) O   8    /814. BD*(1) O  15 - H  48     2.80    0.87    0.044
123. LP (2) O   8    /814. BD*(1) O  15 - H  48    24.28    0.97    0.137

136. LP (1) O  15    /836. BD*(1) O  24 - H  53     3.03    1.00    0.049
137. LP (2) O  15    /836. BD*(1) O  24 - H  53    20.43    0.84    0.118

157. LP (1) F  40    /805. BD*(1) O   8 - H  43     4.21    1.27    0.066
159. LP (3) F  40    /805. BD*(1) O   8 - H  43    19.96    0.97    0.125


tetramer cc
============

162. LP (1) O   8    /***. BD*(1) O  19 - H  57     4.59    0.94    0.059
163. LP (2) O   8    /***. BD*(1) O  19 - H  57    18.31    0.92    0.117

164. LP (1) O  10    /***. BD*(1) O   8 - H  72     3.93    0.98    0.056
165. LP (2) O  10    /***. BD*(1) O   8 - H  72    21.65    0.88    0.124

182. LP (1) O  33    /***. BD*(1) O  10 - H  67     2.03    0.99    0.040
183. LP (2) O  33    /***. BD*(1) O  10 - H  67    20.00    0.84    0.116

203. LP (1) F  50    /***. BD*(1) O  33 - H  62     2.89    1.35    0.056
205. LP (3) F  50    /***. BD*(1) O  33 - H  62    15.84    0.90    0.107


tetramer cc_cycl
=================

162. LP (1) O   8    /***. BD*(1) O  15 - H  62     2.14    0.95    0.041
163. LP (2) O   8    /***. BD*(1) O  15 - H  62    26.51    0.87    0.136

176. LP (1) O  15    /***. BD*(1) O  24 - H  67     3.48    0.97    0.052
177. LP (2) O  15    /***. BD*(1) O  24 - H  67    21.06    0.85    0.120

179. LP (1) O  24    /***. BD*(1) O  33 - H  72     2.88    0.95    0.047
180. LP (2) O  24    /***. BD*(1) O  33 - H  72    25.52    0.84    0.132

182. LP (1) O  33    /***. BD*(1) O   8 - H  57     3.21    0.92    0.049
183. LP (2) O  33    /***. BD*(1) O   8 - H  57    26.29    0.86    0.135
```


[14] F. Weinhold, C. R. Landis, Valency and Bonding A Natural Bond Orbital Donor-Acceptor Perspective, Cambridge University Press, Cambridge, **2005**.

[32] A. E. Reed, L. A. Curtiss, F. Weinhold, Chem. Rev. **1988**, 88, 899-926.
